# Impact of Chronic Obstructive Pulmonary Disease on Long-Term Outcome in Patients with Coronary Artery Disease Undergoing Percutaneous Coronary Intervention

**DOI:** 10.1155/2016/8212459

**Published:** 2016-11-30

**Authors:** Ming Zhang, Yun-Jiu Cheng, Wei-ping Zheng, Guang-Hui Liu, Huai-Sheng Chen, Yu Ning, Xin Zhao, Li-Xiao Su, Li-juan Liu

**Affiliations:** ^1^Department of Cardiology, Beijing Anzhen Hospital, Capital Medical University, Beijing, China; ^2^Department of Cardiology, The Eastern Hospital of the First Affiliated Hospital, Sun Yat-Sen University, Guangzhou, China; ^3^Department of Cardiology, Provincial Clinical Medical College, Fujian Medical University, Fuzhou, China; ^4^Department of Endocrinology, Tongji Hospital, Tongji University, Shanghai 200065, China; ^5^Intensive Care Unit, Shenzhen People's Hospital, The Second Clinical Hospital of Jinan University, Guangzhou, China; ^6^Department of Biostatistics, School of Public Health, Rutgers, The State University of New Jersey, New Brunswick, NJ, USA

## Abstract

*Objective*. The aim of this study was to investigate the association between COPD and major adverse cardiovascular and cerebral events (MACCE) in patients undergoing percutaneous coronary intervention (PCI).* Methods*. 2,362 patients who underwent PCI were included in this study. Subjects were divided into 2 groups: with COPD (*n* = 233) and without COPD (*n* = 2,129). Cox proportional hazards models were analyzed to determine the effect of COPD on the incidence of MACCE.* Results*. The patients with COPD were older (*P* < 0.0001) and were more likely to be current smokers (*P* = 0.02) and have had hypertension (*P* = 0.02) and diabetes mellitus (*P* = 0.01). Prevalence of serious cardiovascular comorbidity was higher in the patients with COPD, including a history of MI (*P* = 0.02) and HF (*P* < 0.0001). Compared with non-COPD group, the COPD group showed a higher risk of all-cause death (hazard ratio (HR): 2.45, *P* < 0.0001), cardiac death (HR: 2.53, *P* = 0.0002), MI (HR: 1.387, *P* = 0.027), and HF (HR: 2.25, *P* < 0.0001).* Conclusions*. Patients with CAD and concomitant COPD are associated with a higher incidence of MACCE (all-cause death, cardiac death, MI, and HF) compared to patients without COPD. The patients with a history of COPD have higher in-hospital and long-term mortality rates than those without COPD after PCI.

## 1. Introduction

Chronic obstructive pulmonary disease (COPD) is a major public health problem that is projected to rank fifth worldwide in terms of disease burden and third in terms of mortality [1]. However, COPD remains relatively unknown or ignored by the public as well as public health and government officials [2]. Most of these studies were retrospective and the diagnosis of COPD was based on clinical history only, without spirometric confirmation, which likely led to an evident underdiagnosis, so the prevalence of COPD is often less than that found in the general population [3, 4]. Elevated cardiovascular disease (CVD) risk is observed and significantly increased in patients with COPD [5]. A diagnosis of COPD increases the risk of CVD and mortality from CVD by an odds ratio (OR) of 2.7 and 1.68 [6, 7]. The degree of airflow obstruction is associated with increased mortality from ischemic heart disease [8]. Decreased pulmonary function is an independent risk factor for CHD mortality [9].

CAD is common in patients with COPD. However, the presence of COPD with coronary artery disease is often not recognized in patients with COPD [10]. Particularly, the impacts of COPD on outcomes after PCI have received less limited attention [11]. It is important to know whether additional short and long-term risks are involved in performing PCI in patients with COPD. The present investigation is to fully assess the impacts of COPD on long-term clinical outcomes in 2,362 consecutive CAD patients undergoing PCI.

## 2. Study Population and Design

We carried out this analysis of acquired data from patients that includes demographic, conventional cardiovascular risk factors, angiographic data, laboratory, and procedural data. In-hospital adverse cardiac events after coronary interventions and hospital stay were recorded and confirmed by reviewing the medical records. Patient medical records were searched for the diagnosis of COPD. The diagnosis of COPD was based on the clinical history or obtained from chart review and recorded as comorbidity in the database. The Institutional Review Board for this study was approved by Beijing Anzhen Hospital.

### 2.1. Clinical Follow-Up

MACCE was defined as cardiac death, MI, target-vessel revascularization (TVR), stroke, and HF. [12] Cardiac death was diagnosed as an unexplained death in which a noncardiac cause had not been identified. The diagnosis of MI was defined as new Q waves, new persistent ST-segment or T-wave changes, or elevated levels of the troponin [13]. The diagnosis of stroke was determined by a neurologist and confirmed on imaging. TVR was defined as any clinically driven revascularization including bypass surgery. HF was defined as any hospitalization in which a suspected diagnosis of HF was the primary reason for admission [12].

### 2.2. Statistical Analysis

Continuous variables were expressed as mean ± SD or median (25th and 75th percentile) and were compared across groups using Student's *t*-test or the Wilcoxon rank sum as appropriate. Categorical variables were presented as counts and percentages and were compared across groups using the chi-squared test or Fisher's exact test as appropriate. The Kaplan-Meier method was used to estimate MACCE-free survival, and the log-rank test was used to test for differences between groups. Cox proportional hazards regression models were used to estimate the association between COPD and long-term MACCE, after adjusting for clinical variables that varied significantly between groups. All tests were two-tailed with a type 1 error rate of 0.05. All analyses were undertaken using SAS 10.5 (SAS Institute, Cary, NC, USA).

## 3. Results

### 3.1. Patient Demographics

We screened a total of 2,362 patients who underwent PCI. Of this sample, 233 patients (9.8%) had a diagnosis of COPD before hospital admission and 2,129 did not have. As shown in Table 1, the patients with COPD were older and more likely to be current smokers and have had hypertension and diabetes mellitus. Various manifestations of CVD were also more prevalent in the personal history of patients with COPD, including MI and HF. Prevalence of white blood cell (WBC) count abnormal was higher in the patients with COPD.

### 3.2. Use of Medications at Discharge

Compared with patients without COPD, patients with COPD were significantly less likely to be prescribed aspirin (87.0% versus 93.0%, *P* = 0.003) and Clopidogrel (43.0% versus 50.0%, *P* = 0.04) after PCI. The use of drugs such as angiotensin-converting enzyme inhibitor and statins did not differ between patients with or without COPD. Conversely, patients with COPD were more likely to be treated with calcium channel blockers (38.0% versus 27.0%, *P* = 0.001) (Table 1).

### 3.3. In-Hospital Cardiovascular Events and COPD

Patients with COPD were more likely to experience adverse events. The patients with COPD were at higher risk of recurrent MI and HF complications. The frequency of MACCE was shown in Table 2. The incidence of HF and MI was significantly higher in patients with COPD; however, the frequency of cardiac death and revascularization was not different between groups.

### 3.4. COPD and Follow-Up MACCE

**Figure 1:**
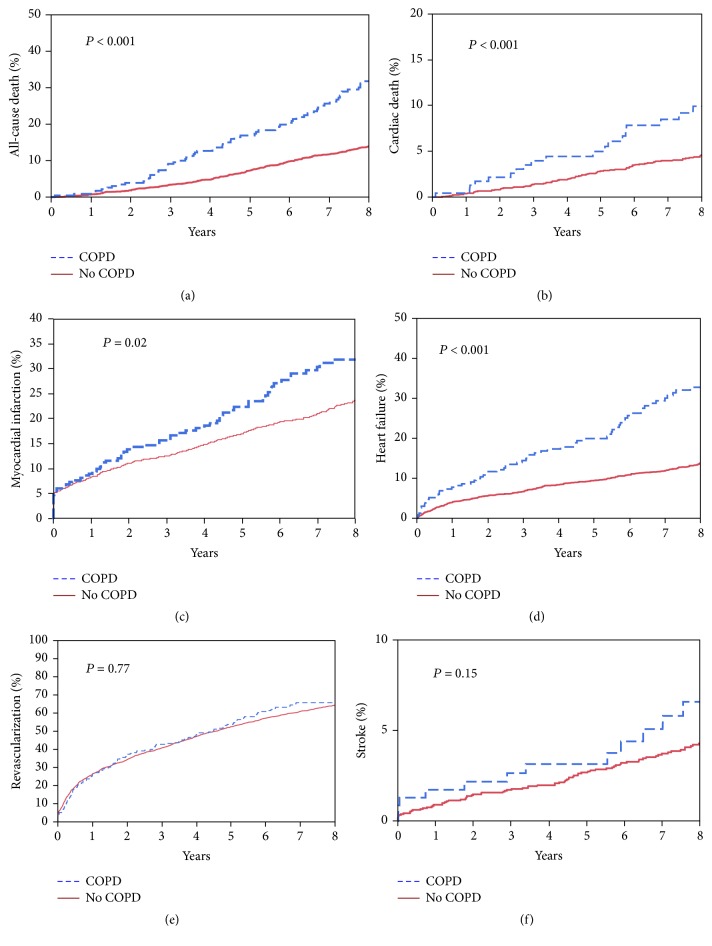
Frequency of adverse cardiovascular events at follow-up in patients with a clinical diagnosis of COPD and no COPD. Unadjusted Kaplan-Meier curves during 8-year follow-up for (a) MACCE; (b) cardiac death; (c) myocardial infarction; (d) heart failure; (e) target-vessel revascularization; (f) stroke.

The relationship between COPD and follow-up MACCE was evaluated using the Kaplan-Meier method. Amongst patients with COPD, estimates for all-cause death (31.79% versus 13.88%, *P* < 0.001), cardiac death (9.92% versus 4.59%, *P* < 0.001), MI (31.87% versus 23.69%, *P* = 0.02), and HF (26.5% versus 32.75%, *P* < 0.001) at 8-year follow-up were significantly higher compared to patients with no COPD (Figure 1).

**Figure 2:**
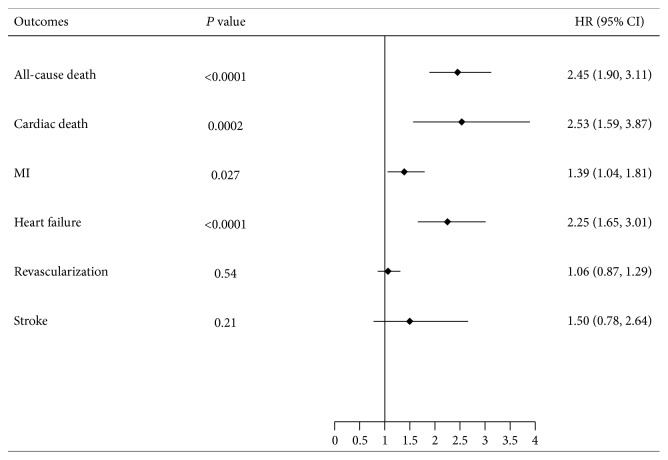
Hazard ratios for MACCE in patients with a clinical diagnosis of COPD and no COPD.

After adjusting for age, gender, diabetes, hypertension, dyslipidemia, current smoking, the history of MI and chronic HF, and a family history of CAD using Cox proportional hazards analysis, the risk of all-cause death (HR: 2.45, *P* < 0.0001), cardiac death (HR: 2.53, *P* = 0.0002), MI (HR: 1.387, *P* = 0.027), and HF (HR: 2.25, *P* < 0.0001) was higher in the COPD group compared with the no COPD group (Figure 2).

## 4. Discussion

The main finding of the present investigation showed that patients with COPD were significantly associated with frequency of in-hospital cardiovascular events including MI and HF and were significantly associated with increased risks of all-cause mortality, cardiac death, and HF during follow-up. Patients with COPD were more likely to sustain a previous MI and had a lower baseline ejection fraction. Despite adjusting for multiple risk factors that may potentially affect clinical outcomes, COPD still remained an independent predictor of long-term cardiovascular events. Our results also showed that the patients with COPD were older and had an increased prevalence of cardiovascular risk factors, which were consistent with previous studies [14–16].

The association between COPD and CAD is well known [5]. In patients with COPD, there is a significantly higher risk of CAD, angina, and MI [6]. The impact of COPD on long-term mortality has been previously investigated. However, most of these studies focused on mortality, but other outcomes such as HF, revascularization, and stroke were not more studied [14, 15]. The present study involved 2362 patients who underwent PCI with diagnosis of COPD confirmed by review of medical records. We fully accessed MACCE, and our study found that these patients with COPD who underwent PCI have an increased risk of adverse cardiovascular outcomes, such as MI, HF, and cardiac death.

COPD could contribute to CAD through multiple pathologic mechanisms, such as systemic inflammation [17, 18] and alterations in pulmonary vessel structure and function, which frequently result in pulmonary hypertension (PH) [19] and increased arterial stiffness independent of cigarette smoke exposure [18–20]. Systemic inflammation as the most important factor, which is defined as the presence of inflammatory/immune response mediators in peripheral blood, is elevated in COPD and involved in the pathogenesis of these disorders. The relationship between COPD, systemic inflammation, and cardiovascular diseases may be especially germane as over half of patients with COPD die from cardiovascular causes [17]. Inflammation has been increasingly recognized to have a role in atherosclerosis particularly in the context of CAD. The chronic systemic inflammation and oxidative stress are important features in COPD [21]. Inflammatory processes in the vessel wall are associated with the progression of atherosclerosis and MI [22]. Metabolic alterations, systemic inflammation [23], and neurohormonal activation frequently occur in patients with COPD [24].

The systemic inflammatory response may cause endothelial injury and vascular dysfunction, although the exact mechanisms remain to be determined [22, 25]. Systemic inflammation is usually evaluated by the biomarkers such as C-reactive protein (CRP) and leukocytes [26]. It has recently been proposed that acute exacerbations of COPD with their associated neutrophil influx may be triggers for acute coronary events [27]. This proposal is supported by studies that have demonstrated that both acute respiratory infection and exacerbations of COPD are associated with a markedly increased incidence of acute coronary events [28, 29]. Our findings suggested that COPD was associated with elevated leukocytes. We found that patients with COPD had an increased prevalence of abnormal baseline WBC count. These results further support that the COPD involves systemic inflammation. Our study demonstrated that the presence of COPD in CAD patients undergoing PCI was associated with adverse outcomes. We should pay more attention to COPD in patients undergoing PCI because they may need closer follow-up and targeted therapeutic interventions. Therefore, pulmonary function tests may be useful and necessary for patients with CAD undergoing PCI.

## 5. Strengths and Limitations 

This is a report with a registry, which contains elements of quality assurance and appears to be adequate for the purpose of the study. The major strength of this study lies on the long-term follow-up data presented, and also fully assessing the impact of COPD on long-term clinical outcomes. However, this study is a nonrandomized, retrospective cohort study of acquired data and has the inherent limitations, including selection and referral biases. There is no enough information on COPD severity and medication. Pulmonary function tests and laboratory data were not available in the database. Therefore, the accuracy of diagnosis and classification of COPD may be criticized. Despite these limitations, we believe our study is the largest to date which attempts to answer whether COPD is associated with higher risk of in-hospital and long-term MACCE following PCI, and our complete patient enrollment provides representative results. Obviously, larger prospective randomized clinical trials will be necessary to confirm these observations.

## 6. Conclusions

COPD is associated with increased mortality and nonfatal clinical events in patients undergoing PCI. Our findings suggest that screening for COPD and establishing its severity in CAD patients undergoing PCI could help in risk stratification to determine the intensity of follow-up and treatment.

## Figures and Tables

**Table 1:** Baseline patient characteristics.

Variable	No COPD (*N* = 2129)	COPD (*N* = 233)	*P value*
*Clinical characteristics*			
Age	63.9 ± 11.3	66.4 ± 12.4	<0.0001
Male, number (%)	1368 (64.3)	149 (64.0)	0.93
Body mass index (kg)	30.1 ± 5.8	30.3 ± 6.8	0.91
Diabetes mellitus, number (%)	547 (25.8)	70 (30.4)	0.01
Hypertension, number (%)	1440 (68.8)	169 (73.5)	0.02
Current smoking, number (%)	279 (14.8)	38 (19.1)	0.04
Hyperlipidemia, number (%)	1459 (80.6)	154 (84.6)	0.37
MI, number (%)	811 (38.5)	99 (43.4)	0.02
F/H CAD, number (%)	606 (36.5)	57 (34.1)	0.55
EF_le40, number (%)	128 (6.0)	19 (8.2)	0.04
CHF, number (%)	190 (9.2)	48 (22.02)	<0.0001
Renal Failure, number (%)	141 (6.6)	20 (8.6)	0.07
*Laboratory characteristics*			
White blood cells, median (*Q*1, *Q*3)	7.5 (6.1, 9.3)	8.1 (6.6, 10.3)	0.0002
Hemoglobin (g/dL), median (*Q*1, *Q*3)	13 (12, 14.1)	12.9 (12, 14.1)	0.17
Platelet (g/l), median (*Q*1, *Q*3)	210 (176, 250)	219 (180, 260)	0.20
BUN	18 (14, 22)	18 (14, 24)	0.27
Cr (mg/dl), median (*Q*1, *Q*3)	1.1 (1, 1.3)	1.1 (1.0, 1.2)	0.47
TC (mg/dl), median (*Q*1, *Q*3)	186 (158, 220)	179 (158, 214)	0.13
TG (mg/dl), median (*Q*1, *Q*3)	147 (102, 208)	141 (93, 190)	0.17
LDL (mg/dl), median (*Q*1, *Q*3)	110 (85, 138)	104 (82, 129)	0.03
HDL, median (*Q*1, *Q*3)	43 (36, 52)	43 (37, 54)	0.13
Left main disease, number (%)	39 (1.8)	5 (2.2)	0.74
Emergent PCI	263 (12)	29 (13)	0.87
DES	549 (26)	53 (22.8)	0.31
*Use of medications at discharge*			
Aspirin, number (%)	1954 (93)	201 (87)	0.003
Clopidogrel, number (%)	1053 (50)	99 (43)	0.04
ACEI/ARB, number (%)	1014 (48)	119 (51)	0.34
CCBs, number (%)	571 (27)	88 (38)	0.001
Beta-blockers, number (%)	1596 (75)	169 (72)	0.36
Statins, number (%)	1585 (75)	182 (78)	0.25

MI, myocardial infarction; F/H CAD, family history of coronary artery disease; BUN, blood urea nitrogen; TG, triglyceride; total cholesterol, TC; HDL-C, high-density lipoprotein-cholesterol; LDL-C, low-density lipoprotein-cholesterol; PCI, percutaneous coronary intervention; DES, drug eluting stent; ACEI, angiotensin converting enzyme inhibitor; ARB, angiotensin receptor blockers. CCBs, calcium-channel blockers.

**Table 2:** Frequency of in–hospital cardiovascular events in patients undergoing PCI.

Variable	COPD (*N* = 233)	No COPD (*N* = 2129)	*P* value
Cardiac death	0 (0)	0 (0)	NA
MI	38 (16.3)	106 (4.6)	0.005
Heart failure	48 (22.0)	190 (9.2)	<0.0001
Revascularization	7 (3.0)	84 (3.9)	0.46

PCI, percutaneous coronary intervention; MI, myocardial infarction.
